# Differential TLR7-mediated cytokine expression by R848 in M-CSF- versus GM-CSF-derived macrophages after LCMV infection

**DOI:** 10.1099/jgv.0.001541

**Published:** 2020-12-17

**Authors:** Torki Alothaimeen, Evan Trus, Sameh Basta, Katrina Gee

**Affiliations:** ^1^​ Department of Biomedical and Molecular Sciences, Queen’s University, Kingston, Canada

**Keywords:** GM-CSF, M-CSF, macrophages, R848, cytokines, TLR7

## Abstract

Granulocyte-macrophage colony-stimulating factor (GM-CSF) and macrophage colony-stimulating factor (M-CSF) play an important role in macrophage (MФ) development by influencing their differentiation and polarization. Our goal was to explore the difference between M-CSF- and GM-CSF-derived bone marrow MФ responsiveness to TLR7-mediated signalling pathways that influence cytokine production early after infection in a model of acute virus infection. To do so, we examined cytokine production and TLR7-mediated signalling at 1 h post-lymphocytic choriomeningitis virus (LCMV) Armstrong (ARM) infection. We found that R848-induced cytokine expression was enhanced in these cells, with GM-CSF cells exhibiting higher proinflammatory cytokine expression and M-CSF cells exhibiting higher anti-inflammatory cytokine expression. However, R848-mediated signalling molecule activation was diminished in LCMV-infected M-CSF and GM-CSF macrophages. Interestingly, we observed that TLR7 expression was maintained during LCMV infection of M-CSF and GM-CSF cells. Moreover, TLR7 expression was significantly higher in M-CSF cells compared to GM-CSF cells. Taken together, our data demonstrate that although LCMV restrains early TLR7-mediated signalling, it primes differentiated MФ to enhance expression of their respective cytokine profiles and maintains levels of TLR7 expression early after infection.

## Introduction

Macrophages (MФ) are phagocytic cells that serve important roles in anti-viral immunity. In response to acute virus infection MФ are excellent producers of cytokines that, if not adequately controlled, can lead to harmful effects. Generally, MФ are activated by a variety of stimuli, which can lead to their polarization into what was originally defined as classically activated M1 MФ or alternatively activated MФ, M2 [1–8]. M1 MФ typically secrete proinflammatory cytokines, such as IL-12, IL-23 and TNF-α [9], and these cells also exhibit an increase of phagocytotic activity, the induction of autophagy and production of nitric oxide [4]. On the other hand, exposure to cytokines such as IL-4, IL-10, or IL-13 shifts MФ polarization towards an M2 phenotype [10].

MФ cultured in granulocyte macrophage colony-stimulating factor (GM-CSF) have been found to be closely related to M1 MФ, based on cytokine expression [6, 7, 11–14]. Monocytes treated with macrophage-colony stimulating factor (M-CSF) differentiate into MФ, which express anti-inflammatory cytokines such as IL-10 and are characterized by enhanced arginase-1 production over nitric oxide [15]. It should be noted that M1 and M2 MФ can share markers such as CD11c, CD11b and MHCII [14, 16–18], reflecting the spectrum of MФ activation [4]. Both M-CSF and GM-CSF play roles in immune responses to virus infection, thus these cytokines may influence the activity of MФ during virus infection, skewing the cytokine response to either a proinflammatory or an anti-inflammatory profile.

Lymphocytic choriomeningitis virus (LCMV) is a natural mouse pathogen, which infects and replicates in MФ [14, 19, 20], as well as in dendritic cells (DCs) [21, 22]. LCMV infection *in vivo* induces M1 activation, as illustrated by the expression of iNOS and YM-1 in splenic MФ after viral infection [23]. Innate immune responses to LCMV infection are led by a variety of pattern recognition receptors (PRRs). Of these, TLR7 is a key endosomal PRR that recognizes ssRNA. With respect to LCMV infection, TLR7 knockout mice (TLR7^−/−^) are unable to induce type I IFN in high levels in response to the acute LCMV-WE strain (LCMV-WE) [24], which indicates the importance of TLR7 during viral infection. Although TLR7^−/−^ mice clear LCMV-Armstrong (LCMV-ARM; model of acute infection), they do not clear LCMV-clone 13 (LCMV-CL13; model of chronic infection) infection [25], indicating a requirement for TLR7-mediated signalling to ultimately activate the adaptive immune responses necessary to clear chronic viral infection. Herein, we focused on the early innate cytokine responses in MФ to investigate how TLR7 responsiveness may be modulated during acute virus infection.

TLR7 is well known to activate IRF signalling and downstream type I IFN production [26–29]. Since early levels of proinflammatory and anti-inflammatory cytokines may dictate the success of the immune response later on, we focused on examining how a relatively short timeframe of LCMV infection (1–6 h) influenced the balance of proinflammatory cytokines versus anti-inflammatory cytokines in M-CSF- and GM-CSF-derived-MФ. We used a model where M-CSF and GM-CSF MФ were primed by infection with LCMV for 1 h prior to stimulation with the TLR7 agonist R848, followed by assessment of TLR7-mediated signalling events and cytokine production. Our data demonstrate that R848 stimulation triggered GM-CSF MФ to produce higher levels of proinflammatory cytokines after viral infection compared to the proinflammatory cytokine levels observed in M-CSF MФ. M-CSF MФ produced high levels of the anti-inflammatory cytokine, IL-10 in response R848 treatment in LCVM-infected cells. Interestingly, relatively high levels of IL-10 were also detected in response to R848 stimulation of GM-CSF MФ. Immediate TLR7-mediated signalling was decreased in response to LCMV infection; however, TLR7 expression was maintained in LCMV-infected cells. Taken together, our data suggest that both M-CSF and GM-CSF MФ are primed by virus infection to enhance TLR7-mediated cytokine induction.

## Methods

### Mice and media

Mice were used as a source for bone marrow-derived MФ (BMDMs). Specifically, 6–8-week-old C57BL/6 (H-2b) purchased from JAX Labs (Bar Harbor, ME, USA) were used. All procedures were carried out in accordance with the guidelines of the Canadian Council of Animal Use and Queen’s University animal ethics procedures. Media [RPMI or Dulbecco's modified Eagle's medium (DMEM), 10 % foetal calf serum (FCS)] for cell culture were purchased from Invitrogen (Ontario, Canada).

### Virus preparation

The LCMV-ARM strain was propagated in baby hamster kidney (BHK) fibroblast cells, in DMEM supplemented with 10 % FCS, originally obtained from F. Lehmann-Grube (Hamburg, Germany). Virus titration was carried out by detection of LCMV-NP by flow cytometry as described by Johnson *et al*. [30]. Media from uninfected BHK cells were used for mock infection conditions.

### Macrophage preparations

Bone marrow was flushed from femurs and tibia with phosphate-buffered saline (PBS). Cells were then resuspended in lysis buffer (1.66 % ammonium chloride) for 5 min to lyse red blood cells. Cells were cultured in six-well tissue culture plates with RPMI supplemented with 10 % FCS (Fisher Scientific, USA), 50 µg ml^−1^ gentamycin and 20 % supernatant from GM-CSF-secreting X63Ag8 cells or 20 % supernatant from M-CSF-secreting L929 fibroblasts. After 3 days, non-adherent cells were removed from both GM-CSF- and M-CSF-conditioned media. BMDMs were generated and the adherent cells were harvested on day 7 post-culture. The phenotypic characteristics of M-CSF or GM-CSF MФ were evaluated by flow cytometry.

### Infection and stimulation of Mф

BMDMs (M-CSF MФ or GM-CSF MФ) were infected with LCMV-ARM [multiplicity of infection (m.o.i.)=3] or mock control media for times ranging from 1 to 6 h. MФ were also infected with LCMV-ARM for 1 h, followed by stimulation with R848 (resiquimod, 1 µg ml^−1^ or 5 µg ml^−1^), and cultured in RPMI with 5 % FCS at 37 °C for various time points. Cell pellets were collected for flow cytometry or Western blotting and cell-free supernatant was collected for enzyme-linked immunosorbent assay (ELISA).

### Flow cytometry analyses

Flow cytometry analyses of M-CSF MФ and GM-CSF MФ were performed after 7 days in culture. For surface marker staining, cells were stained for 20 min at 4 °C with fluorochrome-labelled anti-mouse Abs (Biolegend, USA) specific for CD11b (clone M1/70-PE) and F4/80 (clone BM8-PE/Cy5). Detection of TLR7 was done by intracellular staining; cells were fixed with 1 % paraformaldehyde (PFA) for 20 min, washed twice in PBS and then permeabilized with 0.1 % saponin for 25 min before incubation with PE-conjugated anti-mouse TLR7 (clone A94B10; BD Pharmingen, USA) for 1 h at room temperature. Flow cytometry analysis was performed using the CytoFLEX flow cytometer (Beckman Coulter, USA) and analysed using FlowJo software. The geometric mean fluorescent intensity values are indicated for each histogram.

### Western blotting

M-CSF MФ and GM-CSF MФ were infected with mock or LCMV for 1 h, and then stimulated with R848 for 15 min. Cells were then harvested and pellets were incubated in lysis buffer (1 M HEPES, 0.5 M NaF, 0.5 M EGTA, 2.5 M NaCl, 1 M MgCl_2_, 10 % glyercol, 1 % Triton X-100) with PhosSTOP phosphatase inhibitor (Roche, Switzerland). The Bradford assay (BioRad Laboratories, USA) was used to determine protein concentration. Lysates were subjected to electrophoresis on 10 % polyacrylamide SDS-PAGE and transferred to polyvinylidene difluoride membrane (BioRad Laboratories). Membranes were probed with the following primary antibodies: rabbit anti-phospho-NF-κB (Santa Cruz Biotechnology, USA), anti-phospho-p42/44, anti-phospho-p38 (Cell Signaling Technologies, USA) and secondary antibody : goat anti-rabbit-HRP (Santa Cruz Biotechnology). Membranes were stripped and reprobed with rabbit anti-NF-κBp65, anti-pan p38, or anti-p42/44 (Santa Cruz Biotechnology). All membranes were visualized with Clarity Western ECL Substrate (Bio-Rad, USA) and imaged and quantified using an Alpha Innotech FluorChem HD2 system using AlphaView software version 3.1.0.0. To obtain fold change data, the densitometry of phospho-specific bands was first normalized to respective pan bands and the mock medium control for each of M-CSF and GM-CSF cells was used to calculate the fold change.

### ELISA

ELISA was performed on cell supernatants as per the manufacturer’s instructions using mouse Invitrogen ELISA kits specific for IL-12p70, IL-12/23p40, TNF-α, IL-6 and IL-10 (Thermo Fischer Scientific, USA). Absorbance was read at 450 nm using a BioTek EL800 Plate Reader (Thermo Fisher Scientific) using Gen5 software version 1.04.5. Data were analysed using GraphPad Prism 8 and are shown as the average±sd from a minimum of three different experiments.

### Statistical analysis

Significance was calculated using GraphPad Prism 8. Significance between untreated and R848 stimulated cells was calculated using Tukey’s test corrected for multiple comparisons. To determine significance between mock- and LCMV-infected cells, either two-way analysis of variance (ANOVA) with Tukey’s multiple comparisons test or Bonferroni’s multiple comparison tests were performed. *P≤0.05, **P≤0.01, ***P≤0.001, ****P≤0.0001.

## Results

### M-CSF- and GM-CSF-derived MФ express F4/80 and CD11b surface markers and are readily infected with LCMV

**Fig. 1.**
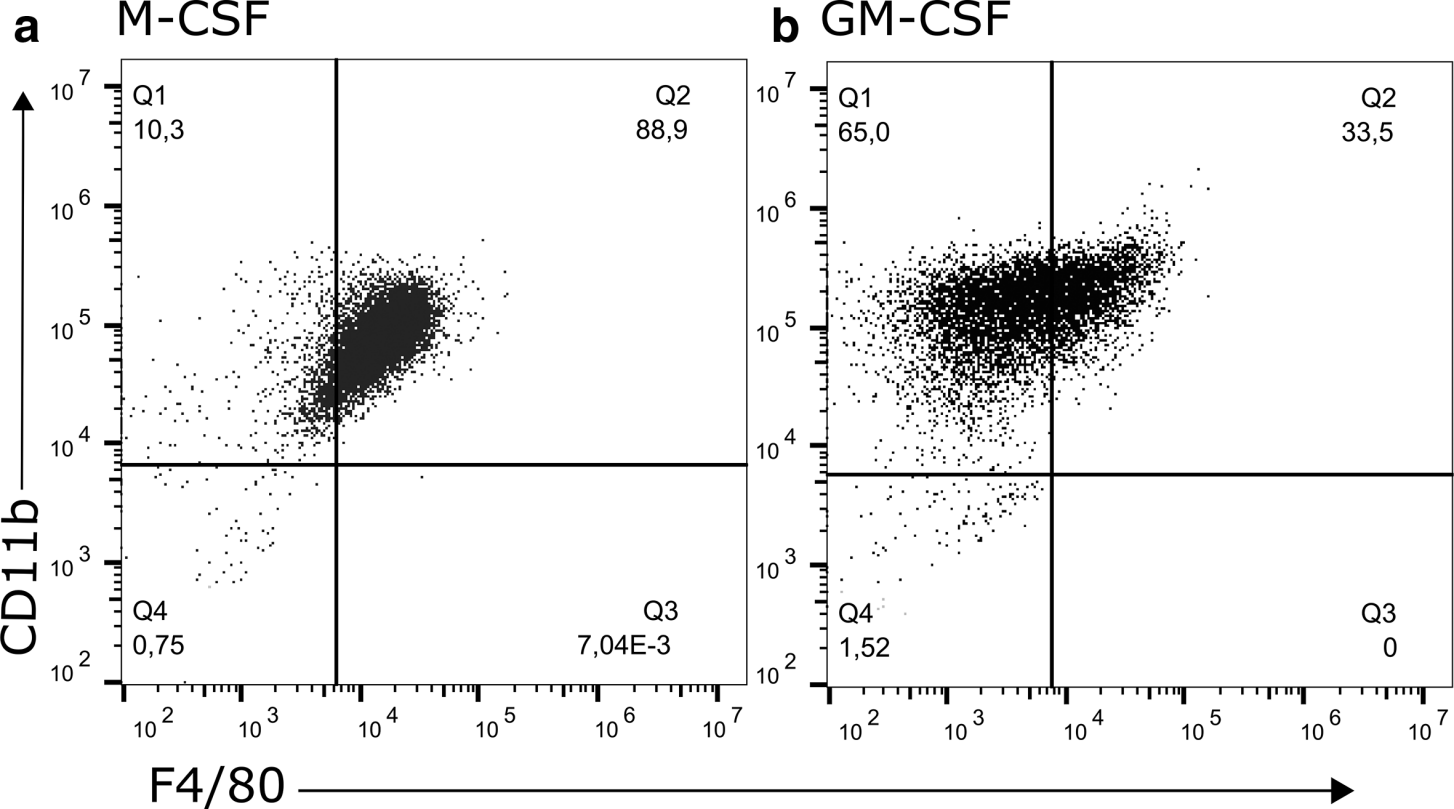
Phenotyping of bone marrow-derived MФ cultured in M-CSF versus GM-CSF. Cells were differentiated with either M-CSF or GM-CSF for 7 days in RPMI with 10 % FBS. On day 7 cells were harvested and co-stained with anti-F4/80 and anti-CD11b antibodies and analysed via flow cytometry. Data shown are representative of three independent biological replicates.

Throughout this study, we used bone marrow-derived cells differentiated in the presence of either M-CSF or GM-CSF, resulting in differentially polarized MФ. Initially, we wanted to confirm that the cells express the well-characterized MФ markers F4/80 and CD11b. As expected, M-CSF-derived cells show higher levels of F4/80 expression compared to GM-CSF-derived MФ (Fig. 1) [12, 14, 16, 18].

**Fig. 2.**
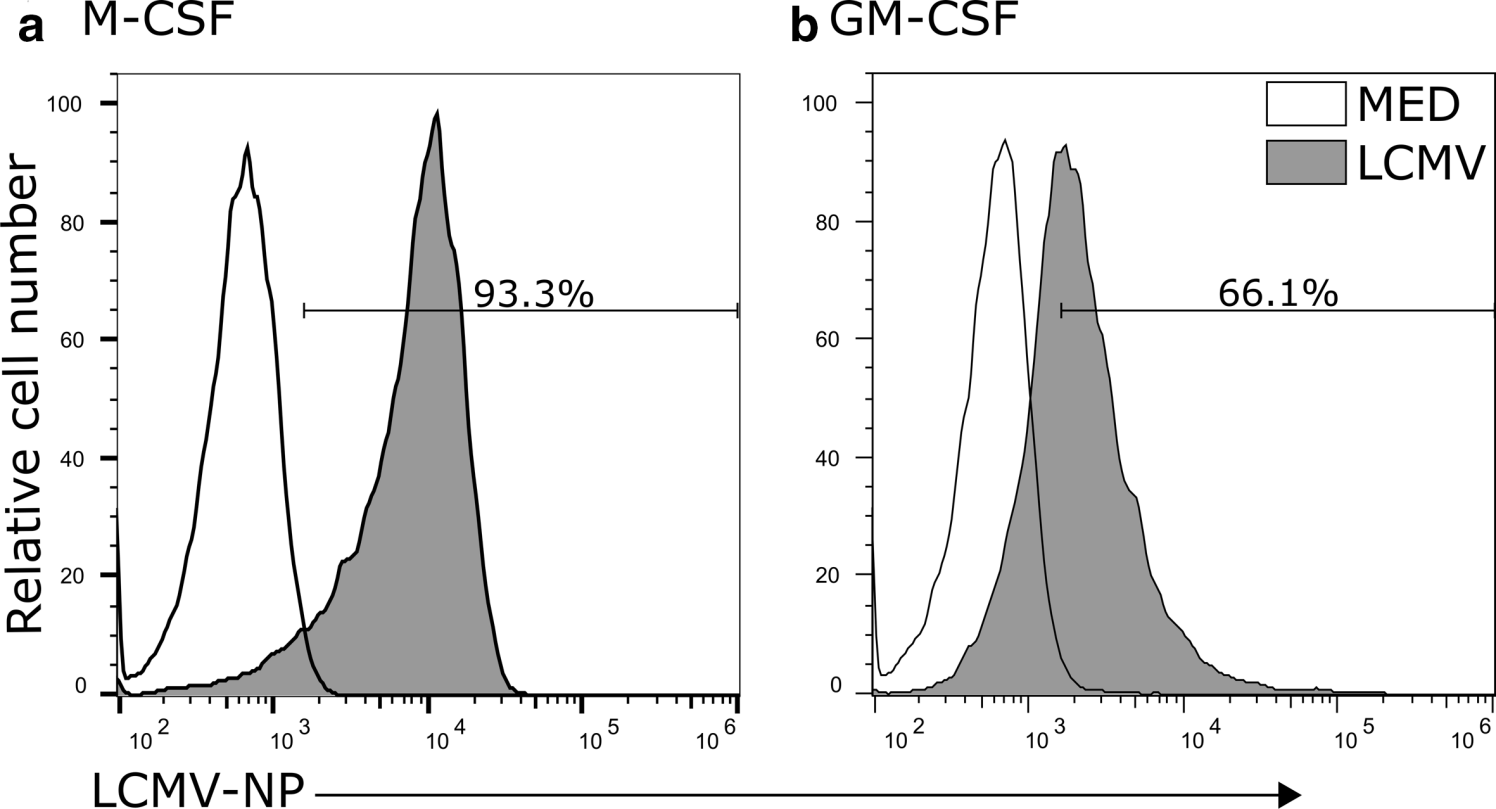
LCMV nucleoprotein expression in infected BMDMs (M-CSF and GM-CSF). *In vitro* LCMV-NP expression in M-CSF- or GM-CSF-induced cells after infection with LCMV. M-CSF (a) and GM-CSF (b) cells were infected at an m.o.i. of 3 for 24 h. Uninfected cells were used in the same labelling protocol to indicate the negative controls (white histogram) and were used to gate on positive NP stained cells (light grey). These data are a representative of three independent biological replicates.

To confirm that M-CSF and GM-CSF cells are permissive to LCMV infection, we cultured the MФ with LCMV-ARM at an m.o.i. of 3, and tested for *de novo* synthesis of LCMV-NP. We incubated the virus with M-CSF MФ and GM-CSF MФ for 60 min to allow for absorption and infection to occur. We then cultured the cells for 24 h at 37 °C to ensure sufficient time for LCMV-NP to accumulate to detectable levels [31, 32]. Following staining for LCMV-NP, flow cytometry was used to measure the percentage of LCMV-NP-positive cells (Fig. 2). The data show that LCMV infected 93.3 % of M-CSF MФ (Fig. 2a; MED MFI: 6950, LCMV MFI: 9600), compared to 66.1 % of the GM-CSF MФ (Fig. 2b; MED MFI: 7640, LCMV MFI: 4550). Thus, the majority of both types of MФ were infected with LCMV.

### M-CSF- and GM-CSF-derived MФ exhibit disparate cytokine responses early post-LCMV infection

**Fig. 3.**
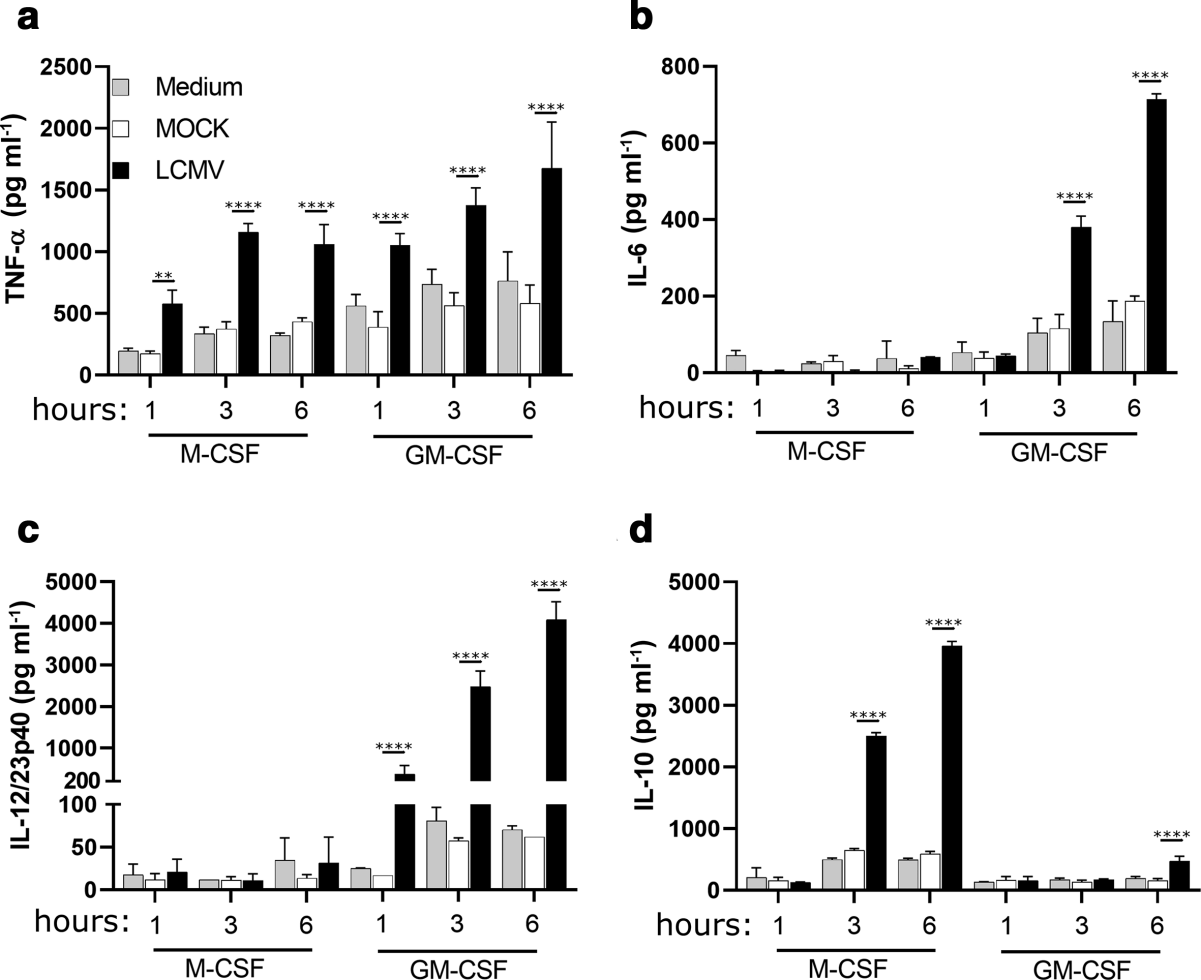
Infection with LCMV causes differential cytokine production in M-CSF- and GM-CSF-derived MФ. Murine bone marrow-derived MФ cultured in M-CSF or GM-CSF for 7 days were exposed to media conditions, mock infection, or LCMV infection for 1, 3, or 6 h before the supernatants were harvested. Supernatants were harvested for cytokine analysis by ELISA: TNF-α (a), IL-6 (b), IL-12/23p40 (c) and IL-10 (d). Data are representative of at least three independent biological replicates and are presented as the mean±sd of three technical replicate wells. Statistical significance for differences in cytokine expression between mock- and LCMV-infected cells was calculated using a two-way ANOVA with Tukey’s multiple comparisons test. *P≤0.05, **P≤0.01, ***P≤0.001, ****P≤0.0001.

We previously examined the influence of LCMV infection on GM-CSF and M-CSF MФ after 6 and 24 h of infection [14]. Herein, we assessed the rapidity of the cytokine response to infection by examining earlier time points of 1 and 3 h and included the 6 h time point as a control (Fig. 3). We confirmed that GM-CSF cells displayed a more inflammatory cytokine profile compared to M-CSF cells. At time points as early as 1 h, GM-CSF MФ exhibited enhanced TNF-α and IL-12/23p40 and by 3 h, enhanced IL-6 expression (Fig. 3a–c). In contrast, M-CSF MФ cells under the same conditions exhibited higher levels of the anti-inflammatory cytokine, IL-10, by the 3 h time point (Fig. 3d). As expected, GM-CSF cells exhibited greater TNF-α, IL-6 and IL-12/23p40 levels compared to mock-treated cells (Fig. 3a–c). At the 6 h time point, LCMV infection induced a low, but significant, amount of IL-10 from GM-CSF cells (Fig. 3d). Taken together, these results indicate that as early as 1 h post-infection, changes in cytokine secretion levels are detectable; therefore, we chose this time point for analysis of TLR7 responsiveness during very early LCMV infection in subsequent experiments.

### LCMV infection primes MФ to secrete cytokines in response to subsequent TLR7 stimulation

**Fig. 4.**
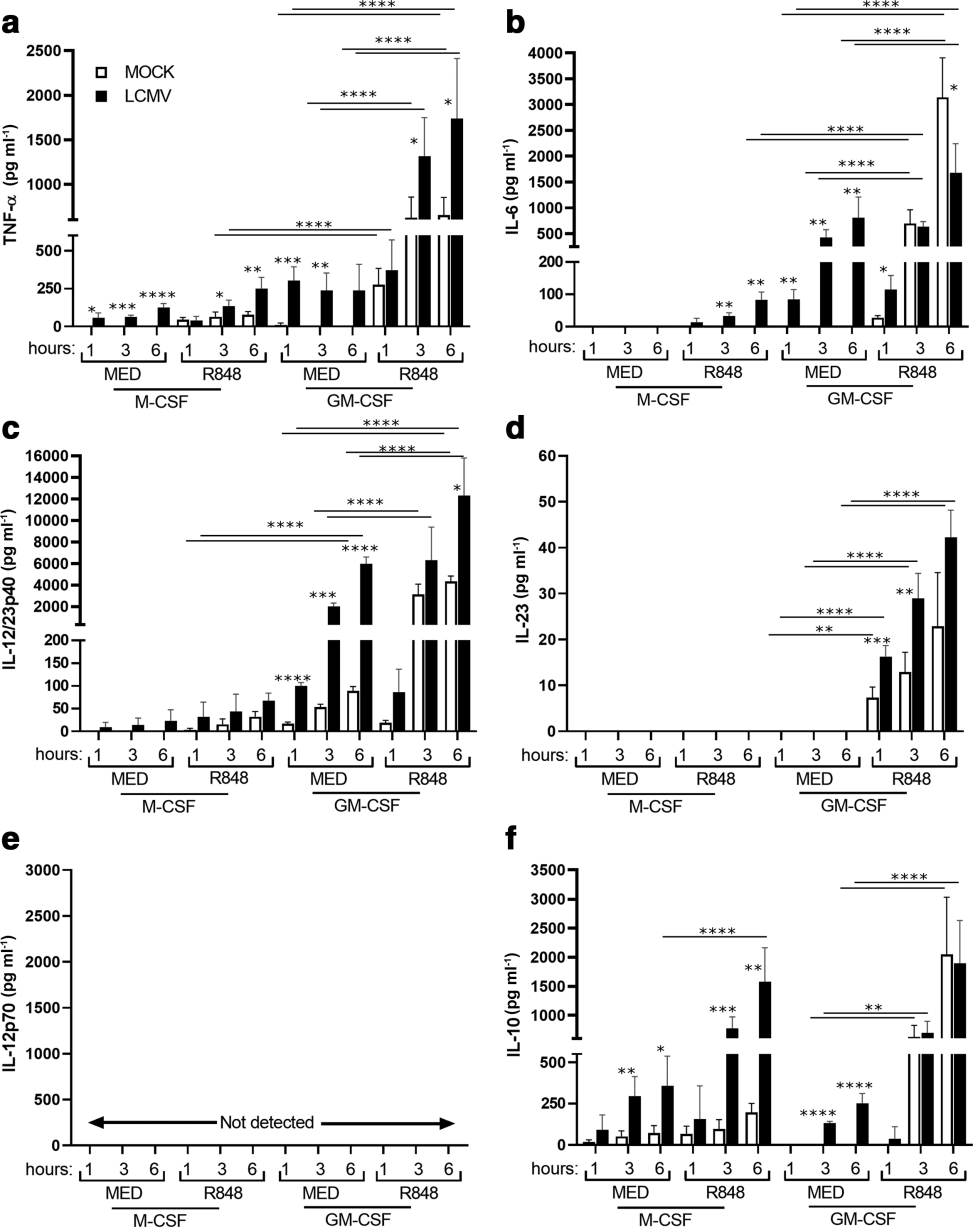
GM-CSF MФ exhibit enhanced proinflammatory cytokine expression in response to LCMV infection and R848 stimulation. GM-CSF MФ and M-CSF MФ were either infected with LCMV (m.o.i.=3) or mock and then stimulated with R848 (1 µg ml^−1^) for 1 h, 3 h and 6 h. GM-CSF and M-CSF cell-free supernatants were collected for the measurement of TNF-α (a), IL-6 (b), IL-12/23p40 (c), IL-23 (d), IL-12p70 (e) and IL-10 (f) by ELISA. Data presented are the mean±sd of three independent biological replicates, each with three technical replicate wells. Statistical significance for differences in cytokine expression between R848 treated and untreated cells was calculated using Tukey’s multiple comparisons test. To determine statistical significance between mock- and LCMV-infected cells, Bonferroni’s multiple comparisons were performed. *P≤0.05, **P≤0.01, ***P≤0.001, ****P≤0.0001.

To evaluate how GM-CSF MФ and M-CSF MФ respond to TLR7 ligation very early after virus infection, differentiated cells were either infected with LCMV or mock for 1 h and then stimulated with R848 for an additional 1, 3 and 6 h. Cell-free supernatants were collected and analysed by ELISA (Fig. 4). Overall, GM-CSF cells expressed higher levels of the proinflammatory cytokines TNF-α, IL-6, IL-12/23p40 and IL-23 in response to R848 in the presence or absence of LCMV compared to M-CSF cells (Fig. 4a–d). Interestingly, IL-12p70 was not detected in any condition (Fig. 4e). However, differential responses to R848 treatment in mock- and LCMV-infected cells with respect to proinflammatory cytokine expression were observed. M-CSF MФ produced low levels of TNF-α in response to virus infection alone and also in response to LCMV infection followed by R848 stimulation (Fig. 4a). However, R848 stimulation of LCMV-infected GM-CSF MФ induced TNF-α to significantly higher levels compared to R848-treated GM-CSF MФ mock controls as well as untreated (MED) and R848-uninfected GM-CSF MФ controls (Fig. 4a). GM-CSF MФ produced significantly higher levels of TNF-α in both mock- and LCMV-infected cells in response after 3 and 6 h of R848 stimulation.

IL-6 exhibited differential responses to R848 stimulation in M-CSF and GM-CSF cells (Fig. 4b). In M-CSF cells, LCMV infection followed by R848 stimulation resulted in the induction of IL-6 expression, but expression of this cytokine was not detected in response to LCMV alone. In contrast, in GM-CSF cells, LCMV infection alone induced IL-6 expression, and in response to R848 alone, these cells produced significantly higher levels of IL-6. LCMV-infected cells at 1 h post-R848 stimulation exhibited significantly higher levels of IL-6; however, these cells exhibited significantly reduced IL-6 production by 6 h post-R848 stimulation compared to mock-infected cells (Fig. 4b). Similar to TNF-α, GM-CSF MФ produced significantly higher levels of IL-6 in both mock- and LCMV-infected cells in response to R848 stimulation (3 and 6 h).

IL-12/23p40 was produced by M-CSF MФ in response to LCMV infection (Fig. 4c), while in GM-CSF MФ, LCMV infection resulted in significantly elevated levels compared to mock control. The combination of virus infection and R848 treatment primed GM-CSF MФ to significantly elevate IL-12/23p40 levels compared to media-treated cells (Fig. 4c). In comparison to M-CSF cells, GM-CSF cells expressed significantly higher levels of IL-12/23p40 in cells infected with LCMV in the presence and absence of R848 stimulation. In contrast to IL-12/23p40, IL-23 was only induced by R848-treated GM-CSF MФ cells, where it was expressed at significantly higher levels compared to media- and mock-infected controls (Fig. 4d).

Expression of the anti-inflammatory cytokine IL-10 was significantly higher in LCMV-infected M-CSF cells compared to mock infection and further stimulation with R848 significantly enhanced the production of IL-10 in LCMV-infected M-CSF cells (Fig. 4f). Surprisingly, R848 and the combination of virus infection and R848 treatment primed GM-CSF MФ to express similar amounts of IL-10 and these levels reached comparable levels to those for LCMV-infected M-CSF cells stimulated with R848 (Fig. 4f). Taken together, these results indicate that GM-CSF cells are more responsive to R848 stimulation with respect to both proinflammatory and anti-inflammatory cytokine expression, while M-CSF cells are more responsive to R848 stimulation with respect to anti-inflammatory cytokine expression.

### R848-mediated signalling is blocked in LCMV-primed cells

**Fig. 5.**
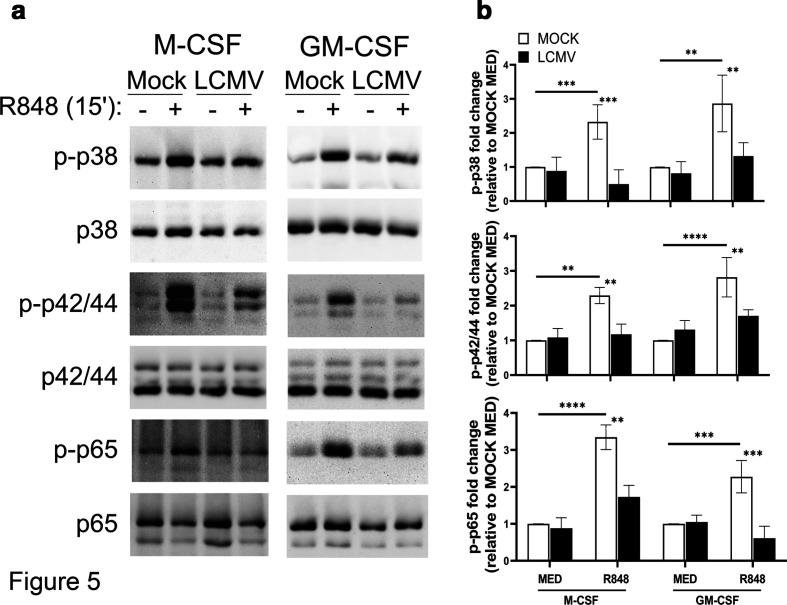
Phosphorylation of p38, p42/44 and NF-kB is decreased in LCMV-infected M-CSF and GM-CSF MФ in response to R848. Murine bone marrow-derived MФ were differentiated in M-CSF- or GM-CSF-enriched media for 7 days before infection. MФ were infected with mock or LCMV for 1 h and then exposed to R848 (5 µg ml^−1^) for 15 min. (a) Cells were then harvested and lysed for immunoblot for phosphorylated (p)-p38, p-42/44 and p-NF-κBp65. Blots were stripped and reproved with pan p38, p42/44 and NF-kB antibodies. Immunoblots shown are representative of at least three independent biological replicates. (b) Densitometry analysis was performed on three independent biological replicates and is graphed as the mean MFI±sd of the fold change compared to mock medium (MOCK MED) controls for M-CSF and GM-CSF. Statistical significance for differences in phosphorylation levels between R848-treated and untreated cells was calculated using Tukey’s multiple comparisons test. **P≤0.01, ***P≤0.001, ****P≤0.0001.

Since we observed differential regulation of cytokine expression in response to LCMV infection and R848 stimulation in the two cell types, we examined whether LCMV infection could influence the TLR7 responsiveness to R848. Differentiated cells were infected with LCMV or mock for 1 h, followed by stimulation with R848 for 15 min. Immunoblots were used to evaluate the phosphorylation and expression of p38, p42/44 and NF-κBp65 (Fig. 5). As expected, mock-infected M-CSF and GM-CSF cells exhibited significant upregulation of phosphorylated p38, p42/44 and NF-κBp65 in response to R848 stimulation, indicating that both cell types are capable of signalling in response to R848.

In LCMV-infected M-CSF and GM-CSF cells, R848-mediated phosphorylation of p38 and p42/44 was significantly reduced compared to mock conditions, although not completely abolished. Similarly, R848-mediated phosphorylation of NF-κBp65 was significantly reduced in LCMV-infected GM-CSF cells and abolished in M-CSF cells. Taken together, these results indicate that LCMV infection dampens TLR7-mediated p38, p42/44 and NF-κB signalling in M-CSF and GM-CSF cells.

### LCMV infection enhances TLR7 expression in GM-CSF cells, while R848 reduces TLR7 expression in GM-CSF and M-CSF cells

**Fig. 6.**
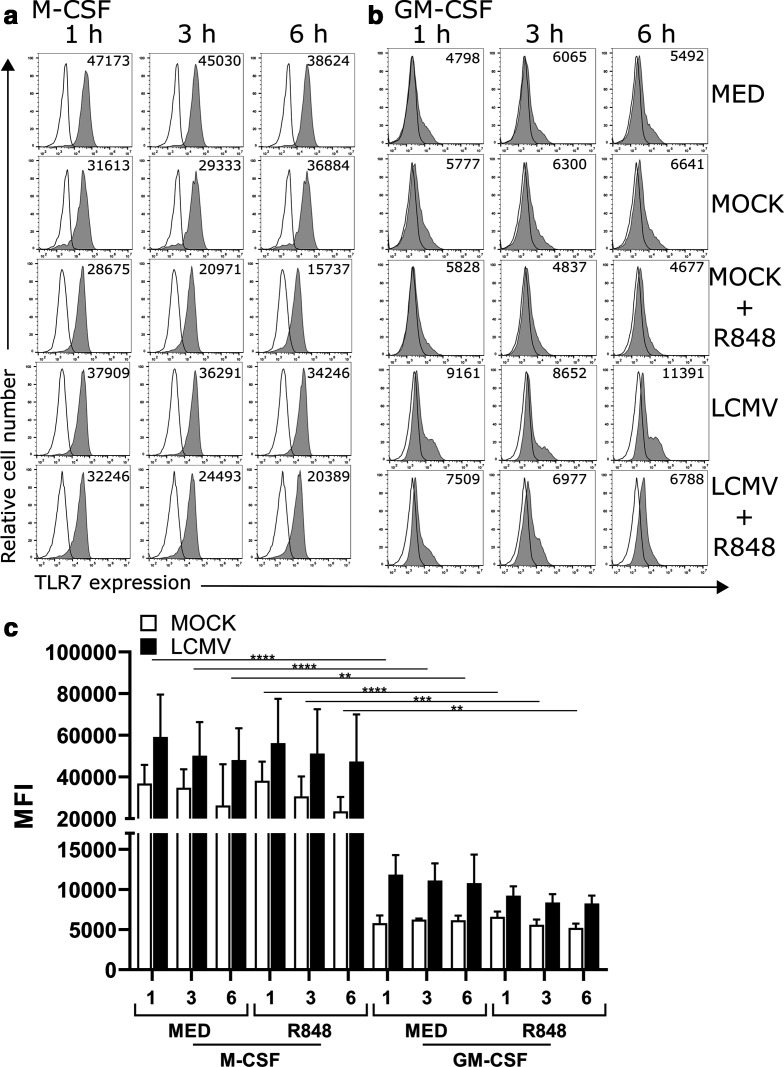
Flow cytometry analyses of TLR7 expression in M-CSF MФ and GM-CSF MФ. M-CSF (a) and GM-CSF MФ (b) cells were fixed and permeabilized prior to intracellular staining for TLR7 (shaded grey histogram). The geometric mean fluorescence intensity (MFI) of TLR7 expression is indicated in the top right corner of each histogram. Isotype controls are indicated by white histograms. Data shown are representative of three independent biological replicates. The average MFI±sd (c) was calculated from three biological replicates. Statistical significance for differences in TLR7 expression between R848-treated and untreated cells was calculated using Tukey’s multiple comparisons test. To determine statistical significance between mock- and LCMV-infected cells, Bonferroni’s multiple comparisons were performed. *P≤0.05, **P≤0.01, ***P≤0.001, ****P≤0.0001.

Since we observed that R848 treatment and the combination of virus infection and R848 treatment modulated cytokine production and TLR7-mediated signalling, we next examined if these parameters could influence TLR7 expression. Therefore, cells were either infected with LCMV or mock, treated with R848 or left in media for 1, 3, or 6 h followed by intracellular staining for TLR7. Analysis of differentiated cells cultured in media or mock control conditions revealed that M-CSF cells expressed significantly higher levels of TLR7 compared to GM-CSF cells (Fig. 6a, b, top 2 rows, and c). M-CSF cells infected with LCMV maintained relatively high TLR7 levels, whereas the levels in M-CSF cells were lower across all time points (Fig. 6a, b, row 4). In LCMV-infected M-CSF and GM-CSF cells, TLR7 expression was higher compared to mock cells (Fig. 6a, b, bottom row), but these differences in expression do not reach statistical significance (Fig. 6c). These results indicate that LCMV infection primes the cells to retain TLR7 expression even in the presence of R848.

## Discussion

In this report, we demonstrate that LCMV infection at an early time point of 1 h can influence the MФ response to further TLR7 activation. We observed that although LCMV infection partially reduces TLR7-mediated signalling in M-CSF and GM-CSF, TLR7 expression levels are maintained. Furthermore, we found that MФ primed by LCMV infection expressed higher levels of cytokines in response to R848 stimulation. Specifically, GM-CSF cells expressed higher levels of the proinflammatory cytokines TNF-α, IL-6, IL-12/23p40 and IL-23, and the anti-inflammatory cytokine IL-10, while M-CSF cells expressed higher levels of IL-10 and only low-to-undetectable levels of TNF-α, IL-6, IL-12/23p40 and IL-23.

Under homeostatic conditions, M-CSF is expressed in circulation and produced by diverse cells, such as endothelial cells, fibroblasts and MФ, while GM-CSF is induced under inflammatory conditions, such as arthritis and atherosclerosis [3–7, 12]. M-CSF and GM-CSF expression is also relevant to the control of ssRNA virus infection, where they are being considered as potential therapeutics for viruses such as HIV and influenza A [3, 33]. For instance, high levels of GM-CSF in the lungs are protective against influenza A infection, and can moderate M1 MФ-mediated inflammation [33].

Typically, GM-CSF and M-CSF have the ability to differentiate MФ into opposing phenotypes, whereby M-CSF MФ tend to have an M2 MФ phenotype, and GM-CSF MФ tend to have an M1 MФ phenotype [12, 14, 16, 17, 20, 23, 34, 35]. Previously, LCMV infection was shown to increase expression of M1 MФ markers such as iNOS and YM-1 expression, indicating that LCMV infection promotes M1 differentiation [23]. However, M2 MФ were represented in a high percentage of total MФ population during chronic LCMV infection [36]. Our data measuring LCMV-NP expression demonstrate that M-CSF MФ exhibited greater levels of LCMV infection and replication compared to GM-CSF MФ; however, despite this, both MФ types exhibited higher levels of R848-induced cytokines in the presence of LCMV infection. This indicates that the virus infection primes the cells for TLR7 responsiveness, supporting the notion that LCMV may promote cytokine induction from pre-existing M1- and M2-polarized cells. In other words, M-CSF MФ and GM-CSF MФ are primed by LCMV infection for enhanced TLR7-mediated induction of anti-inflammatory and proinflammatory cytokines.

Recently, we compared cytokine production by MФ at later time points (6–24 h) in response to LCMV-CL13 and LCMV-ARM, which cause chronic and acute infection *in vivo*, respectively. This study demonstrated that LCMV-CL13 induced lower levels of IL-6 and IL-10 in comparison to LCMV-ARM in GM-CSF MФ and M-CSF MФ, respectively, but induced higher levels of TNF-α in GM-CSF MФ [14]. Overall, GM-CSF MФ responded to LCMV infection by producing a stronger proinflammatory cytokine response compared to M-CSF MФ. In contrast, the anti-inflammatory cytokine IL-10 was significantly induced at high levels by M-CSF MФ [14]. Our current study expands on these results by demonstrating that early after LCMV infection, enhanced sensitivity to TLR7-mediated induction of proinflammatory and anti-inflammatory cytokines from GM-CSF MФ and anti-inflammatory cytokine expression from M-CSF MФ occurs.

We demonstrated that IL-23 was only produced by GM-CSF MФ in response to LCMV infection followed by R848 stimulation, while IL-12p70 was not produced under these conditions. It was unexpected that LCMV infection followed by R848 stimulation did not induce IL-12p70 by MФ *in vitro* because GM-CSF MФ have the capability to express this cytokine in response to LPS stimulation. LPS-mediated TLR4 signalling activates both the MyD88-dependent and MyD88-independent pathways, while R848-mediated TLR7 signalling activates the MyD88-dependent signalling cascade. Therefore, R848 might induce IL-23 expression in response to LCMV infection via a MyD88-dependant pathway but not IL-12p70. Basal IL-12p40 mRNA levels are increased in GM-CSF MФ during differentiation [12] and our data indicate that in GM-CSF MФ, IL-12p40 was induced by LCMV infection alone and R848 alone, and was further enhanced in response to R848 in LCMV-infected cells. Furthermore, given that IL-23 is composed of the p19 and p40 subunits and IL-12p70 is composed of p35 and p40 subunits, it is likely that p19 and p35 expression are differentially regulated in response to LPS versus R848.

Our finding that R848 stimulation alone, as well as LCMV infection followed by R848 stimulation of M-CSF MФ, induced significant levels of IL-10 is supported by a previous study showing that LPS-induced IL-10 was detected in M-CSF MФ [12]. However, this study also showed that LPS-induced IL-10 expression from M-CSF MФ was greater than LPS-induced IL-10 expression from GM-CSF MФ [12]. Interestingly, we show that R848 stimulation enhanced IL-10 production by GM-CSF MФ after LCMV infection as well as mock treatment to comparable levels to those observed in LCMV-infected, R848-treated M-CSF MФ. This highlights the differential response between LPS and R848, and indicates that GM-CSF MФ could be shifted towards an anti-inflammatory or M2 phenotype via TLR7 signalling. Taken together, engagement of TLR7 post-viral infection may play a role in producing anti-inflammatory cytokines such as IL-10, which could lead to a return to homeostasis by controlling the production of proinflammatory cytokines.

MФ express a variety of TLRs that recognize and respond to LCMV-derived PAMPs. Among them, TLR7 is specific to single-stranded RNA, an indicator of viral infection [37]. TLR7^−/−^ mice lack sufficient type I IFN production in response to acute LCMV-WE infection [24], indicating that TLR7 can play a crucial role during viral infection. Furthermore, TLR7^−/−^ mice clear acute LCMV-ARM infection, but not the chronic LCMV-CL13 [25], indicating that TLR7 may play different roles in acute versus chronic viral infection. Upon activation, downstream TLR7 signalling leads to the activation of MAPK molecules and NF-κB, in addition to IRF molecules and type I IFN responses [38–41]. TLR7-mediated MAPK and NF-κB signalling typically lead to the production of proinflammatory and anti-inflammatory cytokines by MФ [42–44]. Activation of p38 is responsible for the induction of the proinflammatory cytokines IL-6, TNF-α and IL-12 in activated MФ, while ERK controls that of IL-10 [44–46].

Interestingly, our data indicate that TLR7-mediated signalling through the p38 and ERK MAPK pathways as well as the activation of NF-κB are partially inhibited in LCMV-primed cells, although, despite this inhibition, cytokine production in response to TLR7 was maintained at robust levels. Maintenance of TLR7 expression in cells primed with LCMV may account for the availability of R848–TLR7 interactions, allowing for increased cytokine expression in M-CSF and GM-CSF cells. Our data suggesting that R848 treatment of mock-infected cells may decrease TLR7 expression agree with other data showing that treatment of peripheral blood monocytes with R848 decreased TLR7 expression [47].

In summary, we demonstrate that R848 triggered GM-CSF MФ to induce high levels of proinflammatory cytokines in the presence or absence of LCMV compared to classical M-CSF MФ. Interestingly, we found that the anti-inflammatory cytokine IL-10 was induced at high levels in response to R848 in GM-CSF MФ, reaching similar levels to those in M-CSF MФ. We also showed that LCMV infection partially decreased activation of TLR7-mediated signalling, while maintaining TLR7 expression. These findings provide new insight into how RNA viruses modulate inflammatory responses, with TLR7 ligation rendering a pronounced cytokine response, suggesting that very early exposure to virus infection functions to prime MФ-led responses.
